# TLR-Mediated Signal Transduction and Neurodegenerative Disorders

**DOI:** 10.3390/brainsci11111373

**Published:** 2021-10-20

**Authors:** Shashank Vishwanath Adhikarla, Niraj Kumar Jha, Vineet Kumar Goswami, Ankur Sharma, Anuradha Bhardwaj, Abhijit Dey, Chiara Villa, Yatender Kumar, Saurabh Kumar Jha

**Affiliations:** 1 Department of Biological Sciences and Engineering, Netaji Subhas University of Technology (Formerly NSIT, University of Delhi), New Delhi 110078, India; adhikarlav.bt.16@nsit.net.in; 2 Department of Biotechnology, School of Engineering & Technology (SET), Sharda University, Greater Noida 201310, India; niraj.jha@sharda.ac.in (N.K.J.); anu.consensus@gmail.com (A.B.); 3 Department of Biotechnology, Delhi Technological University, Delhi 110042, India; vineetgoswami@gmail.com; 4 Department of Science and Engineering, Novel Global Community Educational Foundation, Hebersham 2770, Australia; ankur.sharma7@sharda.ac.in; 5 Department of Life Science, School of Basic Science & Research (SBSR), Sharda University, Greater Noida 201310, India; 6 Department of Life Sciences, Presidency University, 86/1 College Street, Kolkata 700073, India; abhijit.dbs@presiuniv.ac.in; 7 School of Medicine and Surgery, University of Milano-Bicocca, 20900 Monza, Italy; chiara.villa@unimib.it

**Keywords:** TLRs, neuroinflammation, neuroprotection, neurodegeneration Parkinson’s disease, Alzheimer’s disease

## Abstract

A special class of proteins called Toll-like receptors (TLRs) are an essential part of the innate immune system, connecting it to the adaptive immune system. There are 10 different Toll-Like Receptors that have been identified in human beings. TLRs are part of the central nervous system (CNS), showing that the CNS is capable of the immune response, breaking the long-held belief of the brain’s “immune privilege” owing to the blood–brain barrier (BBB). These Toll-Like Receptors are present not just on the resident macrophages of the central nervous system but are also expressed by the neurons to allow them for the production of proinflammatory agents such as interferons, cytokines, and chemokines; the activation and recruitment of glial cells; and their participation in neuronal cell death by apoptosis. This study is focused on the potential roles of various TLRs in various neurodegenerative diseases such as Parkinson’s disease (PD) and Alzheimer’s disease (AD), namely TLR2, TLR3, TLR4, TLR7, and TLR9 in AD and PD in human beings and a mouse model.

## 1. Introduction

Toll-Like Receptors (TLRs) are a family of evolutionarily conserved transmembrane proteins and are classified as membrane-spanning pattern recognition receptor (PRR) proteins [1]. They are mammalian orthologs of *Drosophila melanogaster*’s Toll receptors [1]. TLRs are a part of the innate immune system and are pattern recognition receptors that recognize small molecular motifs from microorganisms called pathogen-associated molecular patterns or PAMPs, and molecules produced endogenously by tissue during inflammation called damage-associated molecular patterns, or DAMPs [2]. TLRs can initiate an acute immune response, and hence stimulate, coordinate, and tune the quality of the adaptive immune response [3].

To date, 19 TLRs have been observed and reported in humans, though many different TLRs are also present in other mammals in different combinations with different functions [4]. The different TLRs are denoted with a different number suffix added to “TLR”. In humans and mice together, we have observed 13 different TLRs: TLR1 to TLR13 (Table 1). Different TLRs are expressed in different species. For example, mice have a gene for TLR10, which does not seem to be expressed due to damage caused by a retrovirus during the course of their evolution [5]. On the other hand, humans do not seem to have genes for TLR11, TLR12, and TLR13, which are present and expressed in mice. Other non-mammalian species also have TLRs which are distinct from those of mammals, such as TLR14 expressed in *Takifugu* pufferfish, which recognizes cell-wall components [6].

Even though TLRs are transmembrane proteins, it is a common misconception that they are all expressed on the cell surface. Some TLRs are expressed inside the cell and are localized within the endosomal compartment of cells, or are present on the membrane of the endosome [7]. TLRs which recognize bacterial and yeast cell products such as peptidoglycans and lipopolysaccharides are present on the cell surface, while oligonucleotide-sensing TLRs are present inside the cell. TLR1, TLR2, TLR4, TLR5, and TLR6 are present on the plasma membrane of mammalian cells [8,9], while TLR3, TLR7, TLR8, and TLR9 are localized within the cells in the endosome [9,10].

TLRs are Type-1 transmembrane proteins containing three structural domains: an extracellular leucine-rich repeat (LRR) motif, a transmembrane domain, and an intracellular cytoplasmic Toll/interleukin-1 receptor (TIR) domain. The pattern recognition of TLRs is brought about by the LRR domain, while the TIR domain interacts with various downstream adaptors and initiates signal transduction [11].

TLRs make use of a variety of adaptors, often in combination with a diverse range of signaling. The most commonly used adaptor is the myeloid differentiation primary response 88 factor (MyD88) [12]. MyD88 is a pivotal molecule in the modulation of the innate immune response because it is the adaptor known to transduce the signal from TLRs by activation of IL-1 receptor associated kinases (IRAKs) via homotypic protein–protein interaction, which eventually leads to the activation of nuclear factor-kappa B (NFκB), mitogen-activated protein kinases (MAP kinases), and activator protein 1 (AP1), making MyD88 a central node for inflammation pathways [13]. MyD88 has two functional domains: the C-terminal TIR domain which allows it to interact with other TIR-containing receptors and adaptors, and an N-terminal death domain (DD), which is involved in the interaction with IRAKs. The DD of MyD88 can independently activate NFκB and c-Jun N-terminal kinase (JNK). The TLR1–TLR2 heterodimer and the TLR2–TLR6 heterodimer make use of TIRAP and MyD88 as adaptors for transduction, while TLR5, TLR7, TLR8, and TLR9 solely make use of MyD88 [14,15].

**Figure 1:**
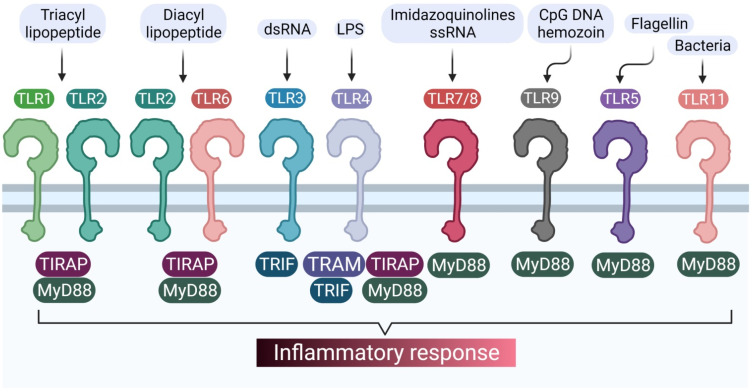
TLRs and their different adaptors in signal transduction pathways.

TLR3 is unique in the sense that it makes use of MyD88-independent signaling pathways, using TRIF as an adaptor [16]. TLR4 is only one of its kind, as it utilizes both MyD88-dependent and MyD88-independent pathways [17]. However, there are a variety of other adaptors which TLRs can make use of, apart from their primary adapters (MyD88 and TRIF), such as adapter protein 3 (AP3) used by TLR9 [15]. Various TLRs and their respective adaptors are represented in Figure 1.

**Figure 2:**
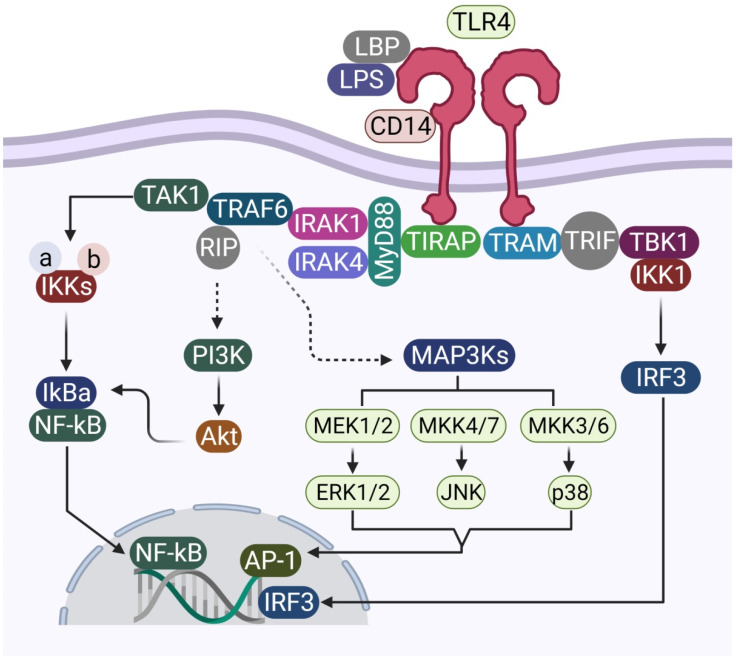
Schematic representation of the various elements involved in MyD88-dependent and MyD88-independent signal transduction, using TLR4 as a model molecule.

The MyD88 pathway, which makes use of MyD88 and TIRAP (Figure 2), activates IRAKs, which, in turn, phosphorylate IκBα protein, which is an inhibitor protein that binds to NFκB, masking its NLS signal and keeping it within the cytoplasm in the inactivated form [18]. Phosphorylation of IκBα results in the release of free NFκB. NFκB is a transcription factor that promotes the transcription of and results in the production of pro-inflammatory cytokines such as TNFα, IL1β, and IL6. The MyD88-independent pathway, which makes use of TRIF as an adaptor, eventually leads to the activation of IRF3, which leads to the production of interferon alpha and beta and other interferon-induced genes. IRF3 plays a crucial role in the production of the antiviral Type 1 interferon [19].

## 2. TLRs in the Nervous System

For quite a long time, the brain was considered to be immunologically privileged, as there seems to be no or very limited immune response within the brain. It made sense, as the brain is a sensitive organ having tissues with poor regenerative capacity. The selective nature of the blood–brain barrier made the belief even stronger. However, over the years, it has been realized that the immunological privilege of the brain is not absolute, and is rather complex, compartmentalized, and region-specific. The “privilege” comes not from the absence of immunological components but a very elaborate and dynamic regulation process. This regulation process is indispensable for damage limitation and mitigation in this sensitive organ [20]. It has also been realized that the cells of the central nervous system (CNS) are quite capable of mounting a dynamic immune response to a variety of stimuli [21].

TLRs play an essential role in this complex regulation within the central and peripheral nervous systems. The expression of TLRs on cells of both the central and peripheral nervous system have been observed and reported [22,23,24,25,26,27,28]. Various cell lines and animal models have been used to study the expression of TLRs in the cells of the nervous system. The expression of TLRs within the nervous systems of mice and humans has been found to be very different, though many of those differences arise from the lack of in vivo studies in humans, with the expression of many TLRs having not yet been studied in the human body. Cell lines, though very helpful in studying TLRs, are not a very accurate representation of in vivo cells owing to the chromosomal aberrations, changes in gene expression, and physiological differences between cancerous cell lines and actual neurons and glia.

In the central nervous system, neurons express TLRs 1 to 9 in both mice and humans. Microglia cells also express TLRs 1 to 9 in both mice and humans. Astrocytes, though known to express TLRs 1 to 9 in mice, only show significant levels of TLRs 1, 3, 4, 5, and 9, while TLRs 2, 6, 7, and 8 have not been reported yet. Oligodendrocytes have also been reported to express only TLR2 and TLR3 in humans, while the other TLRs are yet to be detected. In the peripheral nervous system, the neurons and the resident macrophages express TLRs 1 to 9 in both mice and humans. While the Schwann cells in mice are known to express TLRs 1 to 9, only TLR2 has been very well studied and characterized in human Schwann cells [21,29,30].

In the enteric nervous system (ENS), which is part of the peripheral nervous system of human beings, glial cells are known to express TLRs 2 to 9, while TLR1 seems to be absent in glia. Neurons in the ENS express TLRs 1 to 9 [29]. It is important to note that TLR signaling in neurons does not necessarily use IKKs and NFκB, and may involve the glycogen synthase kinase 3β (GSK3β), Jun N-terminal kinase (JNK), and phosphatidylinositol 3-kinase/protein kinase B (PI3K/AKT) pathways as well.

## 3. TLRs and Alzheimer’s Disease

### 3.1. TLR2

TLR2 recognizes bacterial lipopeptides and peptidoglycans. TLR2 forms heterodimers with TLR1 and TLR6, mediating the response against Gram-positive bacteria and yeast. TLR2 works via the MyD88-dependent pathway, using TIRAP-MyD88 as adaptors and stimulating the production of proinflammatory cytokines using NFκB. TLR2 was found to be upregulated in the microglia surrounding amyloid β (Aβ) plaques, both in human post mortem brains as well as Alzheimer’s Disease (AD)mouse models. Aβ cannot induce an inflammatory response in microglia deficient of TLR2 or in the cortex of TLR2-deficient mice, which acts as further proof that TLR2 has a role in AD [31]. TLR2-deficient AD mouse models show more pronounced cognitive impairment because of greater levels of Aβ proteins and greater white-matter damage [32,33]. The microglial phagocytic response to Aβ is also TLR2-dependent, as Aβ acts as an agonist for microglial TLR2 [34]. Neuronal viability in AD also seems to be affected by microglial TLR2 activation, since neuronal death was shown to be conferred by the release of pro-inflammatory signals by activated microglia [35]. Table 2 summarizes the various possible effects that different TLRs can have on AD.

Neuronal TLR2 can also be implicated in the inflammatory response against Aβ in AD, since TLR2 in neurons is upregulated when neurons are exposed to AD-specific metabolites such as 4-hydroxynonenal (HNE; an AD-related lipid peroxidation product). HNE exposure to neurons also leads to an increase in phosphorylated JNK and cleaved caspase 3, which could indicate AD-related metabolites causing apoptosis in neurons via TLR2 activation [36].

### 3.2. TLR4

TLR4 received attention when an Italian study found that the TLR4 polymorphism is responsible for late-onset of Alzheimer’s disease in the Italian population [37]. An Asp299Gly mutation in TLR4 could confer neuroprotection, since it renders the TLR less responsive to lipopolysaccharides by bringing about structural changes [38]. Another study of post mortem human brains showed TLR4 to be upregulated in the glia surrounding Aβ plaques [39]. Knocking out TLR4 in AD mouse models showed reduced expression of TNFα and the chemokine macrophage inflammatory protein 1β in the cortex [40], though knocking TLR4 out of AD mice led to an increase in the activated microglia, astrocytes, and Aβ protein in the brain [41]. Data from in vitro cultures of microglia revealed TLR4-mediated activation of the microglia in the degeneration of neurons. Microglia seem to play a pro-inflammatory role in AD and have a phagocytic response to Aβ through TLR4, resulting in neuronal cell death. Much like the case of TLR2, neurons respond to Aβ and AD-related metabolites through TLR4, leading to their apoptosis [36]. TLR4 seems to induce a pro-inflammatory response against Aβ aiming to stimulate the activated microglia to clear out the Aβ through microglial uptake. A leading hypothesis regarding the role of TLR4 in AD is that insufficient removal of Aβ leads to its accumulation in the intercellular space, which subsequently activates the microglia and astrocytes which induce apoptosis in affected neurons, causing neuronal cell death.

### 3.3. TLR7 

TLR7 is unique, since this TLR induces neuronal cell death without the activation of glia. More interestingly, the TLR itself is activated by endogenous overexpression of a microRNA, making TLR7′s case very different from that of TLR2 and TLR4. Let-7 is a family of microRNAs (miRNA) which are highly abundant in the brain. The family is known to have a specific GUUGUGU motif in the core sequence of the miRNA. The GUUGUGU motif just happens to match the sequence ssRNA40, a motif in the HIV ssRNA that TLR7 recognizes. It has been found that Let-7; specifically Let-7b, is overexpressed in the neurons of patients with Alzheimer’s disease. This overexpression seems to cause TLR7 to become activated, triggering the production of cytokines such as TNFα, eventually leading to the production of cleaved caspases that cause neurons to undergo apoptosis [10].

Both in vitro and in vivo studies have shown that neither microglia nor astrocytes have a role in neurodegeneration via TLR7 activation. It was also shown that extracellular Let-7 is capable of activating TLR7, essentially showing that neurons that undergo apoptosis release Let-7, which stimulates the TLR7 of neighboring neurons and causes them to undergo apoptosis as well. Beside Let-7, overexpression of any miRNAs with a seed sequence having the GUUGUGU motif such as miR-599 could induce the production of TNFα and negatively affect neuronal viability both in vitro and in vivo [42].

Even in humans, cerebrospinal fluid (CSF) collected from AD patients showed larger amounts of Let-7b as compared with the control group, supporting the hypothesis that Let-7 from dying neurons stimulated the surrounding neurons to undergo apoptosis [10]. RNAs containing the ssRNA40 motif have also been shown to cause neurodegeneration via TLR7 induced by microglia [43]. Apart from TLR7 inducing autophagy, it has also been documented to help with the clearance of Aβ proteins from the system [44]. Knockout models specific for microglia would be needed for deeper studies and better understanding. 

**Table 2:** The various possible effects of different TLRs on Alzheimer’s disease (AD).

TLR	Effects on Alzheimer’s Disease	Reference
TLR2	Activation of microglial, reduces neuronal viability.	[34]
	Is activated in AD patients because of metabolites associated with AD such as HNE, which, in turn, pushes the cell towards apoptosis.	[36]
TLR4	Upregulated in glia surrounding AB plaques.	[39]
	Activation of microglia, reduces neuronal viability.	[41]
TLR7	Activation of TLR7 on neurons can lead to neuronal cell death; does not require activation of glia.	[10]
	Certain RNAs which are overexpressed in AD can trigger TLR7 activation of other surrounding neurons and hence trigger apoptosis in the neurons.	[10]

## 4. TLRs and Parkinson’s Disease

### 4.1. TLR2

Clinical studies have shown that TLR2 is overexpressed in the microglia of patients with Parkinson’s disease (PD), especially in the substantia nigra and the hippocampus region of the brain in the early stages of the disease [33]. In the late stages, TLR2 is upregulated in the striatum [45]. This indicates that TLR2 expression is time-dependent and brain-region-specific. Another set of evidence linking TLR2 with PD is the fact that TLR2 polymorphism is associated with an increased risk of PD. The polymorphisms often lead to changes in the TLR2 promoter, which leads to reduced TLR2 expression [46].

The α-Synuclein has been shown to activate microglia in in vitro cultures via TLR2 [47]. The hypothesis about the role of TLR2 is that it helps microglia clear out excess α-synuclein, but activation of the microglia via the TLR2 pathway induces neurotoxicity. α-synuclein seems to induce a positive feedback loop, which activates the microglia via TLR2 and eventually leads to neurodegeneration, though it is not yet clear why TLR2 shows region- and time-specificity in expression. Table 3 summarizes the effects of TLRs on Parkinson’s disease.

### 4.2. TLR3

TLR3 recognizes double-stranded RNA associated with viruses. Stimulation of TLR3 leads to the production of antiviral interferons such as IFNα and IFNβ via the MyD88- independent TRIF-mediated pathway. It also stimulates natural killer (NK) cells and macrophages to elicit an antiviral response [48]. It is well known that stimulation of TLR3 during the process of neurogenesis causes the process to stop and causes neurodegeneration to occur. The expression of TLR3 in embryonic stem cells stops when neurogenesis begins [49]. Even in adult neurons, stimulation of TLR3 with agonists like poly (I:C) results in growth inhibition of neurons and neurodegeneration. In vivo, injecting postnatal mice with poly (I:C) leads to sensory-motor deficits and fewer axons in the spinal cord [50]. This observation of TLR3 causing neurons to undergo apoptosis led to the hypothesis that TLR3 is involved in virus-associated PD. Parkinson-like symptoms have been observed in several patients of diseases caused by viruses such as HIV [51], Epstein–Barr virus [52], and hepatitis C virus [53]. Even the influenza virus has been reported to cause neurodegeneration [54]. It is possible that activation of TLR3 upon recognition of viral RNA could signal for an immune response, leading to neuronal cell death, or could trigger the neuron to undergo apoptosis, either way leading to neurodegeneration upon activation of TLR3 mediated by viral RNA.

### 4.3. TLR4

TLR4 seems to have both neuroprotective and neurodegenerative roles in the context of PD. TLR4 is upregulated in the post mortem brains of PD patients, suggesting that TLR4 could potentially have a role in neurodegeneration. However, in mouse models of PD, it has been observed that TLR4-deficient mice are more vulnerable to dopaminergic neuronal loss and motor impairment due to α-synuclein overexpression than mice that express TLR4 [55]. At the same time, TLR4-deficient mice are less likely to develop PD symptoms in PD mouse models induced by 1-methyl-4-phenyl-1,2,3,6-tetrahydropyridine (MPTP) [56]. These observations suggest that TLR4 might be neuroprotective in the context of PD, as TLR4 could help in the clearance of toxic aggregates but could cause neurodegeneration in the context of toxin-induced PD, for example in patients who are in contact with agents such as MPTP or rotenone.

Microglial TLR4 has been found to play role in α-synuclein-dependent activation of microglia [57], and microglia activated by α-synuclein tend to downregulate TLR4. It leads to the disabling of a neuroinflammatory positive feedback loop but also reduces the ability of microglia to take up α-synuclein from their environment [47]. Hence, it can be proposed that the balance between the neuroinflammation caused by microglia TLR activation and the endocytosis of α-synuclein would eventually determine if TLR4 plays a neuroprotective role or facilitates neurodegeneration in the case of PD.

### 4.4. TLR9

TLR9 has been observed to be overexpressed in human brain regions such as the substantia nigra and putamen in PD patients and in the brain stem in PD mouse models [58]. Some studies have demonstrated that the activation of TLR9 signaling exacerbates neurodegeneration by inducing oxidative stress and inflammation. Microglia are activated by CpG DNA and induce TNFα and nitric oxide [59]. In a co-culture containing microglia and neurons, the activation of microglia cells by CpG-DNA via TLR9 induced neuronal toxicity, mediated partly through TNF-α [60]. Intracerebroventricular infusions of CpG-DNA caused impairment in spatial memory, microglia activation, and acute axonal damage [61]. Furthermore, intrathecal injection of CpG oligodeoxynucleotide (ODN) induced loss of neurons, axonal injury in the cerebral cortex, and pronounced microglia activation [62].

**Table 3:** Effects of different TLRs on Parkinson’s disease.

TLR	Effects on Parkinson’s Disease (PD)	Reference
TLR2	Upregulated in PD patients, especially in the substantia nigra and in late stages in the striatum.	[45]
	α-Synuclein activates microglia via TLR2.	[48]
	Activation of microglial TLR2 reduces neuronal viability.	
TLR4	Activation of TLR4 can be neurotoxic or neuroprotective, depending on context.	[47]
	It is shown to help clear α-synuclein aggregates in PD.	[47]
	In oxidative stress-induced PD models, TLR4 activation is associated with cell death.	[56]
TLR9	Overexpressed in PD models in regions such as the substantia nigra and the putamen.	[58]
	Its activation leads to an enhancement of oxidative stress for neuronal cells, which exacerbates cell death in PD.	[59]
	Activation of microglia TLR9 reduces neuronal viability.	[59,61]

## 5. Conclusions

However, the current knowledge on the effects and pathways modulated by TLRs in microglia is still modest and further studies are necessary to establish their exact roles in neuropathological events. As evident from the literature, TLRs are involved in a variety of physiological pathways and pathological conditions, and studies that specifically delete these receptors in microglia using models of neurodegeneration could contribute to further clarifying their roles in neuropathological conditions.

It is also evident from the literature that the activation of both the endosomal and plasma membrane receptors control microglial activity and may alter phenotypes, which could control the evolution of neurodegenerative processes. Thus, TLRs could represent potential pharmaco-pathological targets for the development of neuroprotective drugs.

## Figures and Tables

**Table 1:** The different TLRs found in humans and mice, and the ligands that activate those TLRs (TLR10 is not functional in mice, while TLR11 is not detected in humans).

TLR	CD	Ligand	Pathogen Recognised	Localisation
TLR1	CD281	Tri-acyl lipopeptides	Gram-positive bacteria	Extracellular
TLR2	CD282	Di- and tri-acyl Lipopeptides	Gram-positive bacteria; plasmodium	Extracellular
TLR3	CD283	dsRNA	dsRNA viruses	Endosomal
TLR4	CD284	Lipopolysaccharide	Gram-negative bacteria	Extracellular
TLR5	CD285	Flagellin	Motile bacteria	Extracellular
TLR6	CD286	Di-acyl lipopeptides	Gram-positive bacteria	Extracellular
TLR7	CD287	ssRNA	ssRNA viruses	Endosomal
TLR8	CD288	ssRNA; GC rich RNA	ssRNA viruses	Endosomal
TLR9	CD289	CpG DNA	DNA viruses; viral and bacterial DNA	Endosomal
TLR10	CD290	Unknown	-	Extracellular

## Data Availability

Not applicable.
